# Detection of cytogenetic changes and chromosomal aneuploidy with fluorescent in situ hybridization in cytological specimens of oral cancers in Fanconi anemia—Proof of concept

**DOI:** 10.1002/cre2.519

**Published:** 2021-12-02

**Authors:** Bruno Eduardo Silva de Araujo, Eunike Velleuer, Ralf Dietrich, Natalia Pomjanski, Isabela Karoline de Santana Almeida Araujo, Martin Schlensog, Susanne Irmtraud Wells, Josephine Christine Dorsman, Martin Schramm

**Affiliations:** ^1^ Department of Cytopathology Heinrich Heine University Düsseldorf Germany; ^2^ Centre for Child and Adolescent Health HELIOS Klinikum Krefeld Germany; ^3^ German Fanconi Anemia Support Group Eschau Germany; ^4^ Institute of Pathology Heinrich Heine University Düsseldorf Germany; ^5^ Division of Oncology Cancer and Blood Diseases Institute, Cincinnati Children's Hospital Medical Center Cincinnati Ohio USA; ^6^ Department of Clinical Genetics and Human Genetics Amsterdam University Medical Center Amsterdam The Netherlands

**Keywords:** Fanconi anemia, fluorescent in situ hybridization, oral cancer, squamous intraepithelial lesions

## Abstract

**Objectives:**

Fanconi anemia (FA) is a rare inherited DNA instability disorder with a remarkably elevated risk of neoplasia compared with the general population, mainly leukemia and squamous cell carcinoma (SCC). Two thirds of the SCCs arise in the oral cavity and are typically preceded by visible lesions. These lesions can be classified with brush biopsy‐based cytological methods regarding their risk of a malignant transformation. As a proof of concept, this study aims to investigate genetic changes and chromosomal aneuploidy using fluorescent in situ hybridization (FISH) on oral squamous cells derived from FA affected individuals.

**Material and Methods:**

Five FA oral SCC (OSCC) tumor cell lines, one FA OSCC cervical lymph node metastasis as well as tumor‐negative and atypical smears from oral brush biopsies were analyzed with FISH probes covering 5p15.2, *MYC*, *EGFR*, *TERC*, 9q34.1, *CCND1*, 9p21 and centromeres of chromosomes 3, 6, 7, 9, 11, and 17.

**Results:**

OSCC specimens showed gains of all analyzed chromosomal regions. Chromosomal aneuploidy was observed in five of the six OSCC specimens in two multicolor FISH assays with panels of four probes each. Five out of six OSCC specimens displayed a relative deletion of 9p21. Applied on atypical brush biopsy‐based smears, chromosomal aneuploidy was detected in malignant lesions but not in the smear derived from a severe parodontitis.

**Conclusions:**

As proof of concept, FISH was able to detect genetic changes and chromosomal aneuploidy discriminating oral cancer from noncancerous lesions in individuals with FA. This supports its application on oral brush biopsy‐based cytology.

## 1 INTRODUCTION

Fanconi anemia (FA) is a rare inherited bone marrow failure syndrome that in most populations affects approximately one in 200,000 live births (Dufour, 2017) and is characterized by multiple malformations, bone marrow failure, endocrine disorders and a dramatically increased risk of developing hematological malignancies and solid tumors (Dufour, 2017). FA is caused by loss‐of‐function mutations in any of 22 known genes (*FANCA‐W*) leading to spontaneous and inducible genetic instability, hypersensitivity to DNA crosslinkers and oxidative stress, and impaired telomere maintenance and replication (Michl et al., 2016; Nalepa & Clapp, 2018). FA patients especially display a 500‐700‐fold higher risk of head and neck squamous cell carcinoma (HNSCC) compared with the general population, with two thirds of these lesions located in the oral cavity (Kutler et al., 2016).

The treatment of oral squamous cell carcinoma (OSCC) is particularly challenging in FA patients since standard chemoradiation protocols cannot be applied due to the underlying genetic defect in DNA repair pathways that induces DNA‐cross‐linker hypersensitivity also in all normal noncancerous cells. Thus, frequent oral inspections starting at the age of ten is indicated (Fanconi Anemia Research Fund, 2020), in order to discover OSCC at an early stage of development or ideally potentially malignant lesions that likely would progress, where surgery alone is still adequate. Most often, OSCC in FA arises in visible lesions (Velleuer et al., 2020). These are observed to be chronic and frequently syn‐ and metachrone, which hampers repeated controls with tissue biopsies. There is evidence for noninvasive, highly sensitive and specific evaluation of oral visible lesions with brush biopsy‐based oral cytology and DNA ploidy analysis, that identifies oral lesions at risk for malignant transformation (Velleuer et al., 2020). Although, lesions that do not require invasive treatment can be identified with this method, there is still a need to further reduce unnecessary tissue biopsies due to equivocal cytological results that cannot be analyzed for DNA ploidy with DNA image cytometry because of a low number of suspicious cells (<100 cells). Thus, fluorescent in situ hybridization (FISH) emerges as a molecular method with high accuracy to detect cytogenetic changes and chromosomal aneuploidy in cytological specimens (Savic & Bubendorf, 2016) even with a relatively low number of suspicious cells (i.e., 25–50 cells). As the genetic and chromosomal alterations involved in carcinogenesis of FA related OSCCs seems to be similar to sporadic OSCCs (van Zeeburg et al., 2008) we based our search on suitable FISH probes on sporadic OSCCs. Data from sporadic primary OSCCs tumors show that *EGFR* (7p11.2), *PIK3CA* (3q26.32), *CCND1* (11q13.3), and *MYC* (8q24) amplifications, *CDKN2A* (9p21.3) deletions, *TP53* (17p13.1), and *CASP8* (2q33.1) mutations are among the most common aberrations found (Campbell et al., 2018; Cancer Genome Atlas Network, 2015). Moreover, many of the FISH studies on sporadic OSCC use probes for chromosomal regions harboring the above‐mentioned genes or centromere probes (Kokalj Vokac et al., 2014; Lim et al., 2014; Wangsa et al., 2016). This study aims to investigate genetic amplifications, deletions, gains, losses and especially chromosomal aneuploidy in cells from OSCC in FA using FISH. More than half of the worldwide available FA‐related OSCC cell lines and in addition one cytological FA OSCC sample are analyzed. In a proof of concept, these FA OSCCs are compared with normal and some equivocal oral brush biopsy‐based specimens to test the ability of discriminating OSCC from noncancerous oral squamous cells in FA.

## 2 MATERIALS AND METHODS

### 2.1 Cell line cultures and cytological specimens

Cell lines VU1604‐T (RRID:CVCL_XX20), VU1365‐T (RRID:CVCL_XX19), VU1131‐T2.8 (RRID:CVCL_XX18) (P1‐3) from OSCC in FA (van Harten et al., 2019) were provided by Martin Rooimans, Amsterdam University Medical Center. Cell lines CCHMC‐FASCC2 (RRID:CVCL_XX31) (P4) from OSCC in FA (van Harten et al., 2019), and CCHMC‐FASCC1 (P5), derivative of a deidentified OSCC from the tongue of an FA‐affected individual, were generated in the Wells laboratory. The cell lines (Table 1) were cultured at 37°C and 5% CO_2_ as monolayers in 75 cm^2^ flasks. The growth medium consisted of low‐glucose DMEM (31885‐023; Gibco, Invitrogen Corporation), 10% fetal bovine serum (10500‐064; Gibco), and 80 U/ml Penicillin—80 µg/ml Streptomycin (15140‐122; Gibco). Tumor cells of an OSCC in an FA‐affected individual (P6), were aspirated from a cervical lymph node metastasis. The Falcon® tubes containing the tumor cells were vortexed, transferred to a 15 ml tube and centrifuged (Rotina 56; Hettich) for 5 min with 500 g at room temperature. Smears from the sediment were prepared and immediately fixed with Merckofix spray® (Merck) for further analysis with FISH and DNA image cytometry. Archived liquid‐based negative and equivocal oral cytological brush biopsy specimens from FA affected individuals (Velleuer et al., 2020), were uncovered in Xylene for further analysis with FISH and DNA image cytometry.

**Table 1:** Cell lines and tumor cells from oral squamous cell carcinoma in Fanconi anemia

	VU1604‐T	VU1365‐T	VU1131‐T2.8	CCHMC‐FASCC2	CCHMC‐FASCC1	FAOSCC‐MET
Specimen	P1	P2	P3	P4	P5	P6
FA‐type	*FANCL*−/−	*FANCA*−/−	*FANCC*−/−	*FANCA/FANCF*‐deficient	*FANCA*−/−	*NR*
Tumor site	Tongue	Oral mucosa	Floor of mouth	Oral mucosa	Tongue	Lymph node metastasis
Gender	Female	Male	Female	NR	NR	Female

Abbreviation: NR, not reported.

### 2.2 DNA image cytometry

All samples were stained according to Feulgen (Feulgen & Rossenbeck, 1924) for the analysis of DNA ploidy. The processes of cover slipping, de‐staining of the Papanicolaou stain (if necessary), re‐localization of suspicious cells and the measurement of the nuclear DNA content, were performed as previously described (Böcking, 1995; Remmerbach et al., 2009; Schramm et al., 2011). A manual MotiCyte DNA workstation (Motic®) was used, that provides shading and glare correction. Technical and diagnostic recommendations for the measurements, including the definition of a DNA stemline, stemline‐aneuploidy and single cells with a DNA content of >9c (single cell aneuploidy), according to the consensus reports of the European Society for Analytical Cellular Pathology (ESACP) were considered (Bocking et al., 1995; Haroske et al., 1998, 2001). The nuclear DNA‐content in c units was analyzed in approximately 300 nuclei of interest and for internal calibration (2c reference value) in at least 30 normal squamous epithelial cells or granulocytes and plotted in a histogram. Due to the lack of reference cells in the cell line samples, air dried imprint specimens with hepatocytes from rat livers were adopted as an external reference according to the consensus guidelines of the ESACP (Haroske et al., 1998). The imprints were stained according to Feulgen simultaneously for the cell lines to avoid heterogeneity of staining between both samples.

### 2.3 FISH

Four panels of 13 commercially available locus specific (LSI) and centromere (CEP) FISH probes were arranged, including the UROvysion^©^ multicolor probes a separate panel (Table 2). All probes were obtained from Abbott (Abbott Laboratories).

**Table 2:** FISH‐probe panels used in the study

Panel 1	Panel 2	Panel 3	Panel 4
LSI D5S23, D5S721 SpectrumGreen (5p15.2)	CEP 9 SpectrumGreen (9p11‐q11)	CEP 11 (D11Z1) SpectrumGreen (11p11.11‐q11)	CEP 3 SpectrumRed (3p11.1‐q11.1)
LSI *MYC* SpectrumGold (8q24.2)	LSI *TERC* SpectrumGold (3q26)	LSI *CCND1* SpectrumOrange (11q13.3)	CEP 7 SpectrumGreen (7p11.1‐q11.1)
LSI *EGFR* SpectrumRed (7p11.2‐p12)	LSI 9q34 SpectrumAqua (9q34.1)	‐	LSI p16 SpectrumGold (9p21)
CEP 6 (D6Z1) SpectrumAqua (6p11.1‐q11)	‐	‐	CEP 17 SpectrumAqua (17p11.1‐q11.1)

*Note*: Genomic regions and fluorochromes are indicated. 5p15.2 (*Cri‐du‐Chat*), 11q13.3 (*CYCLIN D1*), 9p21 (*CDKN2A*). Panel 4: UROvysion multicolor probe (Abbott Laboratories).

Abbreviations: CEP, centromeric probe; LSI, locus specific probe.

The cytological specimens were investigated with the four panels according to the protocol of Onofre et al. (2008), with the following modifications: After rehydration with an ethanol sequence, the uncovered cytological brush‐biopsy specimens were washed in 2× saline‐sodium citrate buffer for 5 min at 80°C in a water bath. The specimens were then digested by using pepsin 0.2% (Sigma‐Aldrich Co.), for 15 min in a humidified chamber at 37°C and fixed in formalin 1% for 5 min. After dehydration with an ethanol sequence, the FISH probe mix consisting of 3.5 µl Vysis IntelliFISH Hybridization Buffer (Abbott Laboratories), 0.5 µl purified water, and 1 µl probe set was applied to the smears.

The hybridized areas on the specimens were analyzed for atypical nuclei (nuclear enlargement, irregular shape, patchy DAPI staining) using an Axio Imager A1 microscope (Carl Zeiss) equipped with ×63 and ×100 oil immersion objective lenses and an AxioCam MRm video camera (Carl Zeiss). Up to 50 nuclei of interest for each specimen were analyzed by two independent observers and the number of signals for each probe was registered. In addition, the OSCC specimens were analyzed in duplicates. The nuclei of inflammatory cells (e.g., neutrophils) were used as an internal reference for hybridization quality.

### 2.4 FISH evaluation protocol

FISH data were analyzed with descriptive statistics. The mean number of signals per cell and the number of cells with more than two FISH signals were calculated for all cases. A *CCND1* amplification was defined as the *CCND1*/CEP11 ratio ≥2 (panel 3, Table 2), a homozygous (biallelic) deletion of 9p21 was defined as the lack of yellow signals in the UROvysion probe set (Panel 4, Table 2). A relative deletion of 9p21 in a given nucleus was observed if the number of the signals was lower than the number of signals of the centromeric probes for chromosomes 3, 7, and 17 (Zellweger et al., 2006). The use of differentially‐colored FISH probes in a multicolor panel like the UROvysion panel (panel 4, Table 2) or the panel CEP6, 5p15.2, 7p12, and 8q24 (panel 1, Table 2) enabled a combined evaluation of chromosomal gains and losses and the analysis of chromosomal aneuploidy per cell. A cell was defined as chromosomally aneuploid with a gain of two or more of the four probes in panels 1 and 4. A tetrasomic pattern, that occurs both in malignant tumors and euploid polyploidization, was not considered as chromosomally aneuploid to prevent misinterpretation. Tetrasomy was defined as the presence of four copies of the genes in a nucleus. A deviation of ±1 copy of one of the genes, as previously suggested (Schramm et al., 2011), was accepted to consider diagnostic errors. Thus, the following patterns of gene copies were accepted as tetrasomy, regardless of the order: 4‐4‐4‐4, 5‐4‐4‐4, 3‐4‐4‐4.

### 2.5 Ethics Statement

The cytological specimens were leftover samples of our study “Reducing the burden of squamous cell carcinoma in Fanconi anemia” (Velleuer et al., 2020). The participants provided written consent to donate the samples for future research and/or methods development. This procedure was approved by the Western Institutional Review Board® (Puyallup, WA, USA) (number of study: 1139633) and by the ethics committee of the medical faculty of University of Düsseldorf (number of study: 2019‐625).

## 3 RESULTS

### 3.1 DNA image cytometry in OSCC samples and normal controls

**Figure 1:**
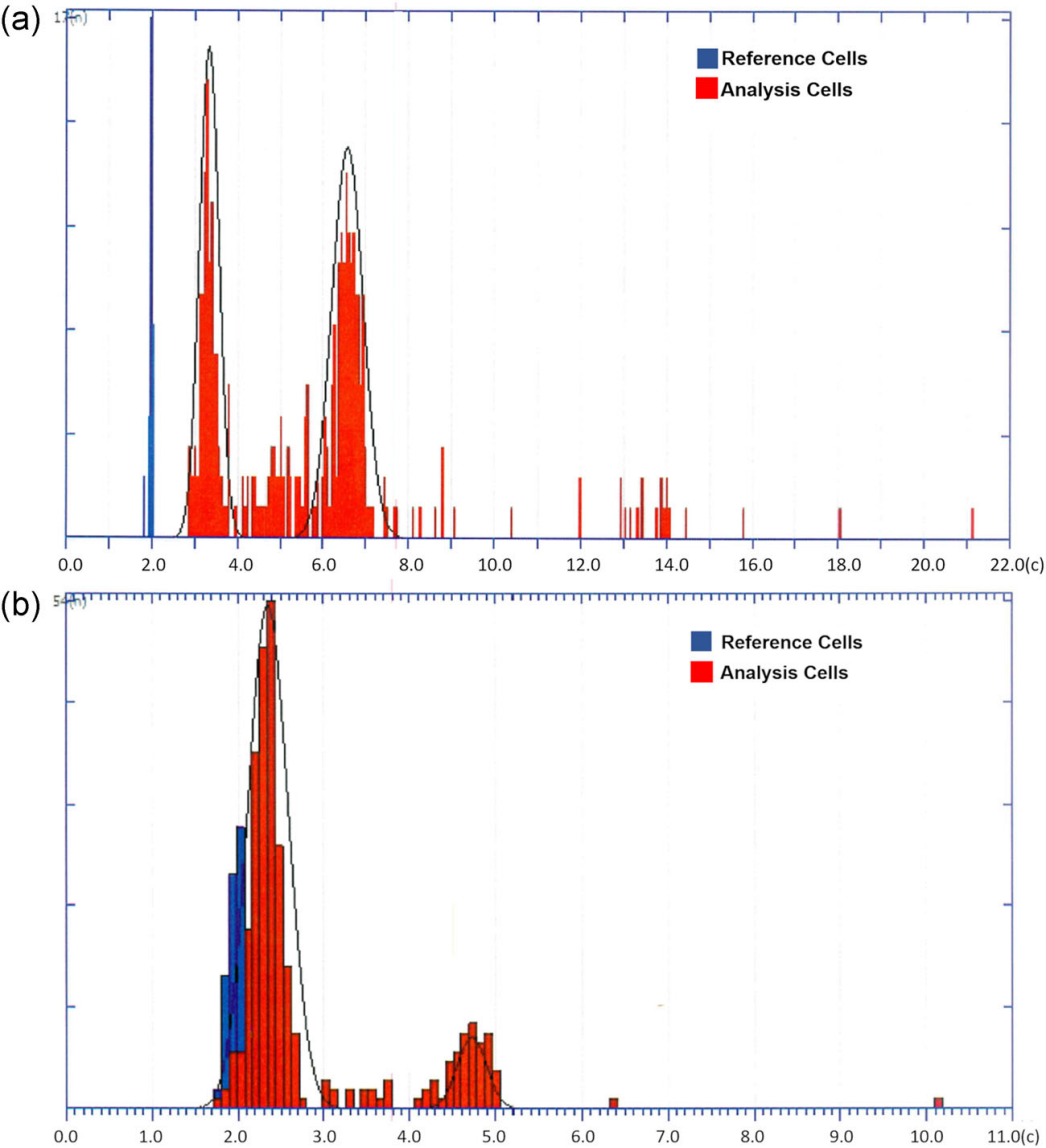
DNA histograms of cell lines VU1604‐T (a) and CCHMC‐FASCC2 (b). DNA‐content in c‐units (*x*‐axis) is plotted against number of cells (*y*‐axis). (a) DNA aneuploidy with two abnormal DNA stemlines (modal values at 3.3c and 6.5c), and a high number of 23 9c exceeding events. (b) DNA aneuploidy with two abnormal stemlines (modal values at 2.33c and 4.77c), and one 9c exceeding event

The DNA ploidy results of the OSCC and tumor negative specimens are reported in Table 3. Figure 1 shows DNA histograms of cell lines VU1604‐T (P1) and CCHMC‐FASCC2 (P4). All OSCC specimens show stemline aneuploidy and single cell aneuploidy in the DNA image cytometry analysis, indicating a malignant transformation. In contrast, both tumor negative samples are euploid. One of them, obtained from a leukoplakia with inflammation at the gingiva, shows an euploid–polyploid histogram with a peri‐diploid and a peri‐tetraploid peak, consistent with euploid polyploidization.

**Table 3:** DNA ploidy results of the OSCC and tumor negative specimens analyzed with DNA image cytometry

Specimen	DNA stemline(s)^a^	>9cEE^b^	DNA ploidy
P1	3.3c; 6.5c	23	Aneuploid
P2	3.59c; 6.9c	2	Aneuploid
P3	4.2c; 6.75c; 7.5c	16	Aneuploid
P4	2.33c; 4.77c	1	Aneuploid
P5	3.46c; 5.67c; 7.4c	11	Aneuploid
P6	3.42c; 7c	13	Aneuploid
N1	1.92c	0	Euploid–diploid
N2	1.92c; 3.84c	0	Euploid–polyploid

Abbreviations: N, negative oral brush‐biopsy‐based cytology; OSCC, oral squamous cell carcinoma; P, OSCC specimens.

^a^ The modal values of DNA stemlines are reported in c‐units.

^b^ >9cEE: number of 9c exceeding events.

### 3.2 FISH copy numbers in OSCC samples and normal controls

**Figure 2:**
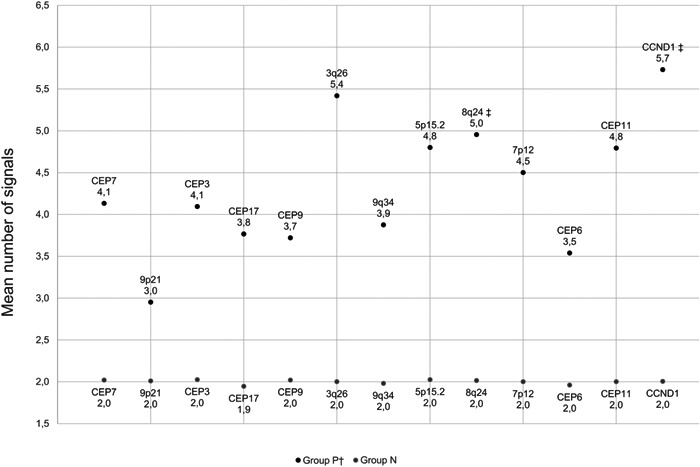
Mean numbers of signals per cell of each FISH probe on OSCC and normal specimens. ^†^Results of P4 were excluded from the analysis. ^‡^Results of P4 and P6 were excluded from analysis

**Figure 3:**
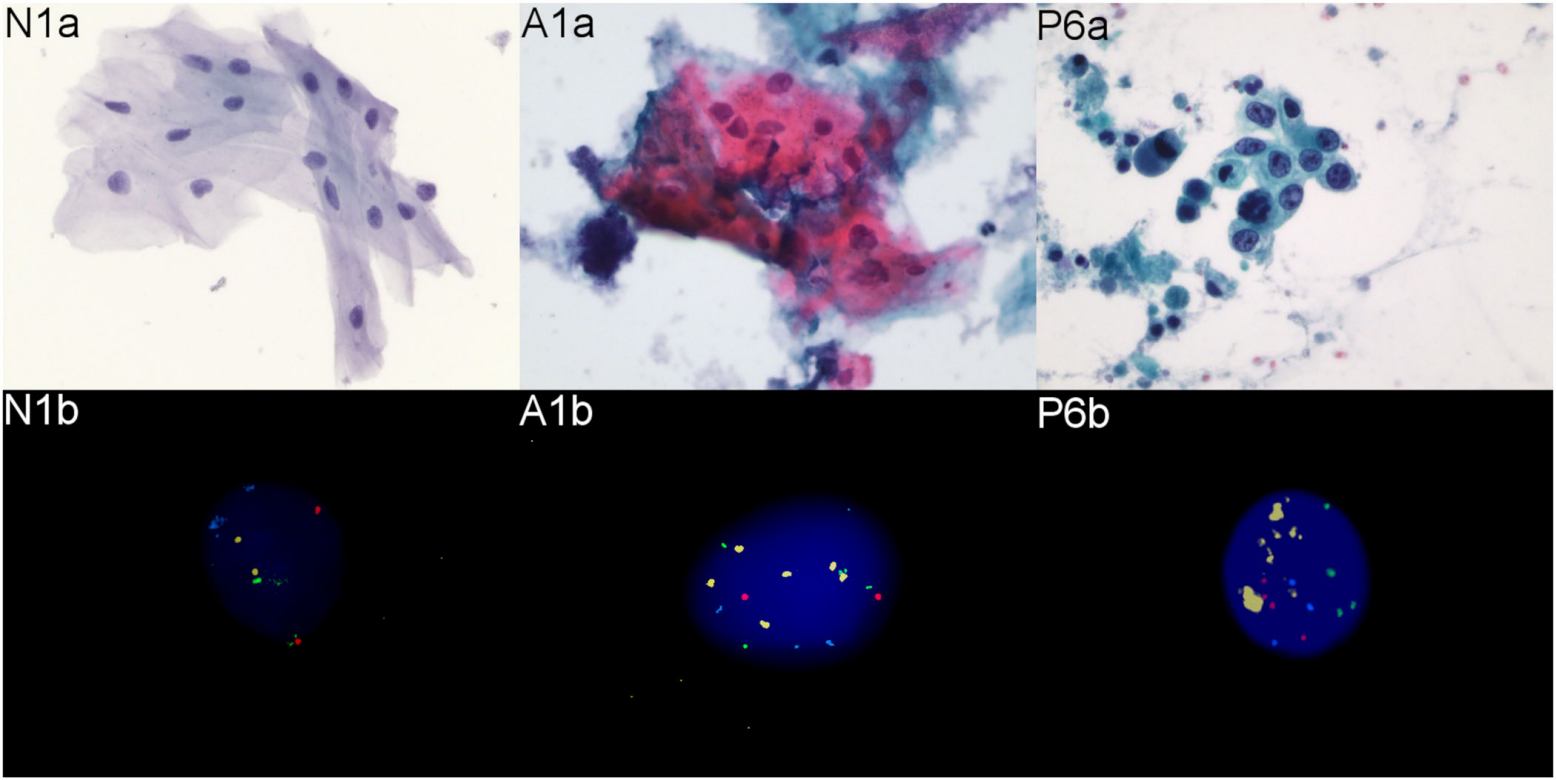
Representative images of the cytological preparations (Papanicolaou stain, original magnification ×40) and fluorescent in situ hybridization (FISH) analyses (original magnification ×63) using probes from panel 1 (5p15.2 (SpectrumGreen), *MYC* (SpectrumGold), CEP 6 (SpectrumAqua), and *EGFR* (SpectrumRed). N1a: normal intermediate squamous epithelial cells in a tumor‐negative brush biopsy‐based specimen; N1b: Corresponding FISH shows disomic pattern of signals. Two SpectrumGreen (5p15.2) signals were counted as one split signal; A1a: Atypical superficial squamous cells with enlarged hyperchromatic nuclei, irregular nuclear contours and enhanced nuclear to cytoplasmic ratio. A1b: Corresponding FISH shows gain of 5p15.2, *MYC* and CEP 6 and disomy of *EGFR*. P6a: Malignant squamous cells from an OSCC cervical lymph node metastasis with nuclear pleomorphism and high nuclear to cytoplasmic ratio; P6b: Corresponding FISH shows gain of *MYC* with clusters and gain of 5p15.2, CEP 6 and *EGFR*

Figure 2 shows the mean number of fluorescent signals per cell of each FISH probe in OSCC (P1‐6) and tumor negative specimens (N1‐2). The results of the near‐diploid OSCC cell line P4 (van Harten et al., 2019), and the results of 8q24 and *CCND1* in the OSCC P6 were omitted from the analysis of the mean numbers to prevent outliers on the mean value. The tumor negative controls show a disomic pattern with a mean of two copies of the respective hybridized chromosomal regions. In contrast, the OSCC specimens display copy number gains, varying from a mean value of three copies per cell of the 9p21 region to 5.7 of the region harboring the *CCND1* gene. OSCC P6 shows a high‐level gain with gene clusters of 8q24 harboring the *MYC* oncogene (Figure 3). Scoring criteria for a *CCND1* amplification in OSCC are heterogeneous in the literature. Correspondingly, we conservatively interpret the *CCND1* amplification with a *CCND1*/CEP11 ratio of 2.72 (Table 4) in OSCC P6 in the current study as low‐level (Blessmann et al., 2013). The mean number of cells that demonstrate a relative deletion of 9p21 in the respective OSCC and tumor negative specimens is shown in Table 4. With the exception of specimen P5, a relative 9p21 deletion at least in 29.5 out of 50 cells was detected in all 5 OSCC specimens. The OSCC specimens do not show any homozygous deletion of 9p21, and the tumor negative controls have always two copies of 9p21.

**Table 4:** Chromosomal aneuploidy, 9p21 deletion and *CCND1* amplification analyzed with FISH in the OSCCs and in tumor negative and atypical oral brush biopsy‐based smears

Specimen	Aneuploid cells, FISH panel 1^a^	Aneuploid cells, FISH panel 4^a^	Relative deletion of 9p21^b^	Ratio *CCND1/*CEP11^c^
P1	49	39.5	46.8	1.21
P2	47.8	48.3	37.3	1.24
P3	34.8	40	29.5	1.02
P4	4.3	4.8	45.3	1.06
P5	36	43.3	6.5	1.04
P6	49.5	39.5	40	2.72
A1	39.5	NR	NR	NR
A2	1.5	NR	NR	NR
A3	34.5	NR	NR	NR
N1	0	0.5	0	0.98
N2	1	0.5	0	1.03

*Note*: 50 cells were analyzed in each case with the exception of 36 cells analyzed in specimen A2.

Abbreviations: FISH, fluorescent in situ hybridization; NR, not reported; OSCC, oral squamous cell carcinoma; P, OSCC specimens; N, normal oral brush biopsy‐based smears; A, atypical oral brush biopsy‐based smears.

^a^ Mean number of aneuploid cells of FISH panel 1 and FISH panel 4.

^b^ Mean number of cells with relative deletion of 9p21.

^c^ Ratio of Cyclin D1 (*CCND1*) and centromere (CEP) 11 copy numbers.

### 3.3 Analysis of chromosomal aneuploidy

We used two FISH panels that each consists of four differentially colored FISH probes to analyze chromosomal aneuploidy in OSCC specimens and normal controls of patients with FA. Out of 50 analyzed cells, all OSCC specimens with the exception of OSCC P4 have high mean numbers of 34.8 to 49.5 aneuploid cells in FISH panel 1 and 39.5 to 48.3 aneuploid cells in FISH panel 4 (Table 4). The tumor negative controls show at maximum one chromosomally aneuploid cell.

Figure 3 illustrates the cytomorphology and ploidy analysis with FISH panel 1 on an OSCC (P6), a tumor negative sample (N1) and an equivocal brush biopsy‐based cytological specimen (A1). As a proof of concept, chromosomal aneuploidy was analyzed in three equivocal oral brush biopsy‐based specimens with FISH panel 1 as well. The mean numbers of aneuploid cells are reported in Table 4. Specimen A1 was cytologically classified as suspicious for malignancy, as previously reported (Velleuer et al., 2020) and displayed a high number of aneuploid cells with FISH panel 1. The lesion at the tongue was excised and histologically a microinvasive SCC arising in a squamous carcinoma in situ was diagnosed. Specimen A2 displayed abnormal regenerating cells, cytologically classified as atypical. The number of aneuploid cells analyzed with FISH was low. Correspondingly, the excisional biopsy at the mandibular gingiva displayed a severe chronic and active ulcerating parodontitis. Specimen A3 obtained from the gingiva adjacent to a large SCC of the lower jaw in an overall palliative situation, was initially classified as cytologically negative, but the clinically requested DNA image cytometry displayed stemline aneuploidy, indicating a malignant transformation as part of a genetically altered cancerogenic field. This is in concordance with the high number of aneuploid cells analyzed with FISH panel 1.

## 4 DISCUSSION

Although early OSCC detection and treatment is known to improve survival for the general population (Amit et al., 2013), most OSCCs in FA are diagnosed at late stages (Kutler et al., 2016). Brush‐biopsy based cytology including the analysis of DNA ploidy of visible, oral lesions is not invasive, has a high negative predictive value and the ability to detect OSCCs and potentially malignant lesions in FA at a noninvasive or early stage (Velleuer et al., 2020). However, due to the lack of a sufficient number of atypical cells in some of those specimens, an alternative method to DNA ploidy testing is necessary to assist the microscopic cytological diagnosis. FISH requires very few atypical cells for the detection of chromosomal changes and aneusomy. The main limitation of this methodology in our proof‐of‐concept study is the lack of a cutoff determination for the diagnosis of aneusomy and its validation in a larger cohort. The main advantage is the transferability of the approach to oral brush biopsy‐based cytology which is noninvasive and well‐tolerated. All FISH probes showed high mean copy number gains ≥ 3 in the OSCC specimens, but no gains in the normal controls. This is consistent with data from “The Cancer Genome Atlas Network,” that report copy number alterations in most HNSCC (Cancer Genome Atlas Network, 2015). To avoid interference with euploid polyploidization in inflammatory conditions and tissue repair (Biesterfeld et al., 1994) that is, in oral graft versus host disease following a hematological stem cell transplantation (Grein Cavalcanti et al., 2015), the mean copy numbers should be different to the value four for the FISH application in a multicolor probe set. It is important to consider euploid polyploidization, that has 2^*n*^ chromosomal sets (e.g., tetrasomy), in scoring algorithms for a multicolor FISH assay applied to equivocal cytological specimens (Schramm et al., 2011) to reduce the risk of false‐positive diagnosis regarding a malignant transformation. While copy number gains and relative loss of 9p21 were frequently observed in the OSCC specimens, the amplification of *CCND1* or a high‐level gain of *MYC* could be detected with FISH only in specimen P6, that was provided by a patient with a highly aggressive OSCC with multiple metastases. This finding is in line with a previous study, in which aberration in *CCND1* number was described as a predictor of cervical lymph node metastasis (Myo et al., 2005).

The analysis of DNA ploidy is a valuable tool for detecting a malignant transformation of oral squamous epithelial cells in FA (Velleuer et al., 2020) and therefore we used this method to be compared with the FISH results. The analysis of DNA ploidy correlates with the chromosomal aneuploidy detected with multicolor FISH. In general, FISH is commonly used in equivocal cytology of different organs, like lungs or bile ducts (Levy et al., 2007; Schramm et al., 2011), but was not tested previously on equivocal oral cytology, especially in the context of FA. Using DNA image cytometry, we observed DNA aneuploidy in all OSCC specimens, but not in the tumor‐negative controls. These results were consistent with the analysis of chromosomal aneuploidy with the two 4‐probe multicolor FISH panels, which showed high mean numbers of cells with chromosomal aneuploidy in the OSCC specimens with the exception of OSCC P4. The application of multicolor‐FISH on brush biopsy‐based cytology was investigated with the panel 1 FISH probe set (Table 4) on three cytologically equivocal specimens. Herein the two cytological specimens obtained from a cancerogenic field and a minimal invasive OSCC showed much higher numbers of chromosomally aneuploidy cells compared with the specimen brushed from a severe parodontitis with tissue regeneration.

In conclusion, as a proof of concept, our study has shown the potential for the application of several FISH probe panels for the detection of genetic changes and chromosomal aneuploidy linked to oral carcinogenesis in OSCC specimens from individuals affected by FA. High numbers of chromosomally aneuploid cells or cells with relative loss of 9p21 are detected in most OSCC specimens with multicolor FISH panels but not in brush biopsy‐based oral cytology with normal or regenerating oral epithelial cells. However, clinical application will require a determination of cutoff for the minimal number of chromosomal aneuploid cells or cells with relative deletion of 9p21, and validation in a cohort of FA affected individuals.

## CONFLICT OF INTERESTS

Dr. Silva de Araujo and Dr. Schramm report grants from German Fanconi Anemia Support Group, grants from the German Federal Ministry of Education and Research, grants from Austrian Fanconi Anemia Support Group and grants from Swiss Fanconi Anemia Support Group, during the conduct of the study. Dr. Velleuer reports grants from German Fanconi Anemia Support Group and grants from Fanconi Anemia Research Fund, during the conduct of the study. Until July 2019, Ralf Dietrich was managing director of the German Fanconi Anemia Support Group and reports grants from Fanconi Anemia Research Fund, during the conduct of the study. Dr. Pomjanski and Miss de Santana Almeida Araujo report grants from German Fanconi Anemia Support Group, grants from Austrian Fanconi Anemia Support Group and grants from Swiss Fanconi Anemia Support Group, during the conduct of the study. Dr. Wells has a patent NMR based metabonomics to diagnose genome instability pending, a patent Upregulation of gangliosides in Fanconi anemia patients pending, and a patent IRAK as a target for head and neck cancer licensed to Kurome. Dr. Dorsman and Dr. Schlensog have nothing to disclose.

## AUTHOR CONTRIBUTIONS

*Conceptualization, data curation, visualization, investigation, methodology, formal analysis, writing–original draft, and writing–review and editing*: Bruno Eduardo Silva de Araujo. *Data curation, project administration, and writing–review and editing*: Eunike Velleuer and Ralf Dietrich. *Investigation, methodology, and writing–review and editing*: Natalia Pomjanski. *Software, formal analysis, and writing–review and editing*: Isabela Karoline de Santana Almeida Araujo. *Investigation, methodology, and writing–review and editing*: Martin Schlensog. Resources, writing–review and editing: Susanne Irmtraud Wells and Josephine Christine Dorsman. *Conceptualization, supervision, data curation, funding acquisition, investigation, project administration, methodology, formal analysis, writing–original draft, and writing–review and editing*: Martin Schramm.

## Data Availability

Research data are not shared.
